# Identification and characterization of an abundant lipoprotein from *Methylacidiphilum fumariolicum* SolV

**DOI:** 10.1007/s00203-023-03603-y

**Published:** 2023-06-12

**Authors:** Changqing Liu, Federica Angius, Arjan Pol, Rob A. Mesman, Wouter Versantvoort, Huub J. M. Op den Camp

**Affiliations:** Faculty of Science, Department of Microbiology, Radboud Institute for Biological and Environmental Sciences, Radboud University, Nijmegen, The Netherlands

**Keywords:** Acidophile, Cell wall, Lipoprotein, Methanotroph, *Methylacidiphilum*, Peptidoglycan, Verrucomicrobiota

## Abstract

Bacterial lipoproteins are characterized by the presence of a conserved N-terminal lipid-modified cysteine residue that allows the hydrophilic protein to anchor into bacterial cell membranes. These lipoproteins play essential roles in a wide variety of physiological processes. Based on transcriptome analysis of the verrucomicrobial methanotroph *Methylacidiphilum fumariolicum* SolV, we identified a highly expressed lipoprotein, WP_009060351 (139 amino acids), in its genome. The first 86 amino acids are specific for the methanotrophic genera *Methylacidiphilum* and *Methylacidmicrobium*, while the last 53 amino acids are present only in lipoproteins of members from the phylum Verrucomicrobiota (Hedlund). Heterologous expression of WP_009060351 in *Escherichia coli* revealed a 25-kDa dimeric protein and a 60-kDa tetrameric protein. Immunoblotting showed that WP_009060351 was present in the total membrane protein and peptidoglycan fractions of *M. fumariolicum* SolV. The results suggest an involvement of lipoprotein WP_009060351 in the linkage between the outer membrane and the peptidoglycan.

## Introduction

The bacterial cell envelope is a complex, multi-layered structure that shapes the cell and provides protection from their external environment (Silhavy et al. 2010). The cell envelope of diderm (Gram-negative) bacteria consists of three distinct layers: an inner cytoplasmic membrane (IM, a symmetrical phospholipid bilayer), an aqueous periplasm containing a thin layer of peptidoglycan, and an outer membrane (OM, an asymmetrical bilayer containing phospholipids and lipopolysaccharides). This lipid asymmetry gives the outer membrane the ability to act as a selective barrier, effectively keeping ions, proteins and other substances where they are needed, preventing them from diffusing and blocking external toxic compounds from entering the cell.

Bacterial lipoproteins are a subset of membrane proteins containing a conserved lipid-modified cysteine (Cys) at the N-terminus that allows them to be inserted into the membrane. Specifically, lipoproteins have an N-terminal signal peptide sequence of about 20 residues. This signal peptide contains a conserved 'lipobox' motif Leu-(Ala/Ser)-(Gly/Ala)-Cys at its C-terminal region (Braun and Wu 1994). After cleavage, the N-terminal amino acid of the mature protein is a Cys, which is modified with diacylglycerol and fatty acyl chains (Narita and Tokuda 2016). This modification allows anchoring hydrophilic proteins into the inner or outer membrane where they perform various important functions, including cell wall metabolism, cell division, adhesion, nutrient uptake and antibiotic resistance (Nakayama et al. 2012).

Precursor-lipoproteins are synthesized in the cytoplasm with a N-terminal signal peptide and then translocated across the IM through the Sec pathway (Pugsley 1993) or the Tat pathway (Narita and Tokuda 2016; Shruthi et al. 2010). The conversion of pre-lipoproteins to mature lipoproteins involves sequential reactions including lipidation and cleavage of the signal peptide. More specific, the N-terminal part of the pre-lipoprotein with the signal sequence is inserted into the IM, while the remaining part of the pre-lipoprotein is oriented towards the periplasmic space. Subsequently, diacylglycerol is attached to the conserved cysteine of the lipobox (see above) by the lipoprotein diacylglyceryl transferase (Lgt), generating an acylated pro-lipoprotein. This modification is a prerequisite for recognition by the lipoprotein signal peptidase II (LspA), which cleaves the signal peptide, generating diacylglycerol apo-lipoprotein. Next, a third acyl chain is added to the conserved cysteine by lipoprotein N-acyltransferase (Lnt), yielding the mature lipoprotein (Nakayama et al. 2012; Narita and Tokuda 2016). Lgt and Lsp are conserved in all bacterial species, whereas Lnt has only been identified in proteobacteria and actinomycetes (Nguyen et al. 2020). After maturation, the lipoproteins are localized to either the IM or the OM of Gram-negative bacteria depending on the sorting signals.

Nowadays, a large number of available genome sequences confirm that bacterial lipoprotein genes are widespread and represent 1–3% of the total genes in a (meta)genome (Babu et al. 2006; Sutcliffe et al. 2012). Even though lipoproteins are known to be important for both cellular physiology and immunology, much remains to be discovered. In particular, as new strains are isolated and genomic data are analyzed, more novel lipoproteins are being identified and need to be characterized. *Methylacidiphilum fumariolicum* SolV, as a representative of the verrucomicrobial methanotrophs, thrives in harsh volcanic environments and plays a vital role in mitigating methane emissions in these geothermal ecosystems (Pol et al. 2007; Schmitz et al. 2021). Currently, more than a dozen verrucomicrobial methanotrophs have been isolated and divided into two genera *Methylacidiphilum* (optimum pH 2–2.7, temperature 50–55 °C) (Dunfield et al. 2007; Islam et al. 2008; Pol et al. 2007;) and *Methylacidimicrobium* (optimum pH 1–3, temperature 30–44 °C) (Sharp et al. 2014; van Teeseling et al. 2014a). Up to date, *M. fumariolicum* SolV is the most studied amongst them (Schmitz et al. 2021). This strain is an autotroph as CO_2_ can be assimilated using the Calvin-Benson-Bassham cycle ((CBB, ribulose-1,5-bisphosphate carboxylase/oxygenase (RuBisCO)) (Khadem et al. 2011) and able to fix N_2_ gas (Khadem et al. 2010). Besides CH_4_, H_2_ can also be used as the only energy source, making this strain a real ‘Knallgas’ bacterium (Mohammadi et al. 2017). Although we have a preliminary understanding of its physiological properties, the proteins involved in cell structure and mechanism remain unknown. Analysis of the genome and transcriptomic data of *M. fumariolicum* SolV grown under different conditions (Mohammadi et al. 2017) revealed in all sets a highly expressed putative lipoprotein (protein ID: WP_009060351). This protein appeared to be highly conserved in *Methylacidiphilum* and *Methylacidimicrobium* species. Herein, we show the bioinformatic analysis of this lipoprotein, as well as expression in *E. coli*, localization studies using immunoblotting and discuss possible functions of this putative lipoprotein.

## Materials and methods

### DNA extraction, strain, plasmid and primers

The DNA of *M. fumariolicum* SolV was extracted using the PowerSoil DNA Isolation Kit (MO BIO Laboratories Inc., Carlsbad, CA, USA). The DNA was used as a template for amplifying the gene *MFUM_0397* encoding the putative lipoprotein WP_009060351. The PCR product was cloned using *Nco*I and *EcoR*I into the corresponding sites of pET-30a to create pET-30a-*MFUM_0397*-His-tag and pET-30a-*MFUM*_0397 according to standard procedures. The strain *E. coli* Rosetta™ 2(DE3) strain was used as the host to express protein WP_009060351, and *E. coli* DH 5ɑ was used for DNA manipulation. All constructed plasmids were verified by both colony PCR and Sanger sequencing. Plasmids and primers are compiled in Supplementary Table S1.

### Purification of the heterologous expressed His-tag lipoprotein

The plasmid pET-30a-*MFUM_0397*-His-tag was transformed into the competent cells *E. coli* Rosetta™ 2(DE3). The cells were grown in 600 ml of LB medium, with appropriate antibiotics at 37 °C and 200 rpm. When the cell density reached an OD_600_ of 0.6–0.8, the cultures were induced with a final concentration of 0.1 μM isopropyl-β-d-thiogalactopyranoside (IPTG), and incubated at 28 °C for an additional 16 h. Cells were collected (5000 × g, 4 °C,15 min) and washed twice using Tris buffer (50 mM Tris pH 8.0, 100 mM NaCl). Cell pellets were resuspended with 20 mL cell lysis buffer (50 mM Tris pH 8.0, 100 mM NaCl, 2 mM EDTA and 0.5% Triton X-100 and 4 M urea). A final concentration of 100 µg/mL lysozyme and protease inhibitor cocktail (Sigma) was added, and cells were homogenized using the Polytron tissue grinder homogenizer until all clumps were disrupted. Cells were broken by passing them through a French pressure cell (120 MPa, three times; American Instrument Company, Silver Spring, MD, USA), cooling the cell suspension to 4 °C after each pass. The lysate was centrifuged at 10,000 × g, 4 °C for 15 min and the resulting clarified supernatant was collected and loaded onto a Ni–NTA (GE Healthcare) column that was equilibrated with binding buffer (50 mM Tris pH 8.0, 100 mM NaCl, 4 M urea). After washing, bound proteins were eluted with a gradient elution buffer (50 mM Tris pH 8.0, 100 mM NaCl, 4 M urea, 500 mM imidazole). The purified lipoprotein was dialyzed overnight against dialysis buffer (0.2 M Tris pH8.0, 0.5 M NaCl, 5% glycerol and 5 µM EDTA) at 4 °C, and analyzed by SDS-PAGE.

### Purification of the heterologous expressed (no His-tag) lipoprotein from *E. coli* with Triton X-114

Lipoprotein purification was performed according to the method of Parra et al. (2010). Briefly, the plasmid pET-30a-*MFUM_0397* was transformed into the competent cells *E. coli* Rosetta™ 2(DE3). Cells were collected and disrupted by a French pressure (120 MPa, three times), with cooling the cell suspension to 4 °C after each pass. Then 1% Triton X-114 was added to the lysate and the suspension was incubated with gentle mixing overnight at 4 °C. The lysate was then centrifuged at 10,000 × g at 4 °C for 20 min, and the supernatant was carefully collected and incubated at 37 °C for 20 min to allow partitioning. Next, a centrifugation step, 5 min at 10,000 × g at room temperature was included to improve phase separation. Then the aqueous phase was removed and transferred to a new tube. The detergent phase was collected in a fresh tube, diluted with cold buffer (50 mM Tris– HCl, 100 mM NaCl, 0.25% N-lauroylsarcosine pH 7.4) to reduce the final detergent concentration to 0.3%. The proteins were analysed by SDS-PAGE.

### NPN uptake assay and diSC_3_(5) release assay

To investigate if lipoprotein WP_009060351 expression in *E. coli* can affect inner membrane and outer membrane stability of the host, diSC_3_(5) (dipropylthiadicarbocyanine iodide) release assays and NPN (1-N-phenylnapthylamine) uptake assays were performed in *E. coli* according to Konovalova et al. (2016).

### Membrane proteins, lipoproteins and peptidoglycan extraction from *M. fumariolicum* SolV

*M. fumariolicum* SolV was cultured in a continuous bioreactor under methane limitation. The medium composition and chemostat operation were as described previously (Schmitz et al. 2020), without the addition of H_2_. Cell were harvested from the chemostat, washed twice with phosphate-buffered saline (PBS) and resuspended in 10 mM HEPES, pH 7.4. The cells were lysed by three passes through a French press (120 MPa). The lysed cells were centrifuged at 10,000 × g for 10 min at 4 °C to remove cell debris and non-lysed cells. The membrane fraction was collected by ultracentrifugation of the supernatant at 100,000 × g for 1 h at 4 °C.

The lipoprotein fraction was extracted using 1% Triton X-114 as described above for *E. coli*. Peptidoglycan isolation was performed according to the methods of Leduc et al. (1989), and Schaub and Dillard (2017). Briefly, DNase and RNase (final concentrations 0.1 mg per ml) were added, and cells were disrupted by three passages through a chilled French pressure cell operated at 120 MPa. After removing unbroken cells, the cell envelopes were pelleted by ultracentrifugation for 30 min at 300,000 × g and suspended in 10 ml of 9% NaCl, mixed with an equal volume of 8% SDS, and incubated for 30 min at 100 °C. Boiling SDS was used to remove cellular components that were not covalently bound to the peptidoglycan. After standing at room temperature overnight, the sample was centrifuged at 30 °C for 30 min at 400,000 × g. The pellet (purified peptidoglycan) was suspended in 1 ml of water, then washed by four cycles of resuspension and centrifugation, and finally resuspended in 1 ml of 10 mM sodium phosphate buffer pH 8. 20% β-mercaptoethanol was added to dissolve the cross-linked components from the purified peptidoglycan by cleavage of the disulphide bonds, and incubated for 30 min at 37 °C.

### SDS-PAGE and MALDI-TOF MS

Samples obtained as described above were denatured by incubation of the proteins for 5–10 min at 100 °C with 4 × sample buffer (250 mM Tris–Cl pH 6.8, 8% SDS, 40% glycerol, 20% β-mercaptoethanol, 0.02% bromophenol blue). SDS-PAGE was performed on 4–16% gels in running buffer. After proteins separation, they were visualized with Coomassie brilliant blue (G250).

Bands cut out from the SDS-PAGE gels were prepared for MALDI-TOF MS analysis according to the method of van Teeseling et al. (2014b). Briefly, the gel pieces were washed alternately in acetonitrile and 50 mM ammonium bicarbonate, then reduced in 10 mM dithiothreitol (DTT) solution and alkylated in 50 mM 2-chloroacetamide and 50 mM ammonium bicarbonate. This was followed by overnight digestion in 50 mM ammonium bicarbonate using 12.5 ng/ml trypsin. Finally, peptides were extracted from the gel pieces using a 1: 1 mixture of 0.1% trifluoroacetic acid and acetonitrile. For MALDI-TOF MS, samples were applied to MALDI target plates using 4-cyano-hydroxycinnamic acid as a matrix and analyzed using a Bruker Microflex® MALDI-TOF MS instrument. Proteins were identified using the MASCOT search tool (Matrix Science, London, United Kingdom) with the "*M. fumariolicum* SolV" predicted proteins database from the Genoscope website (https://mage.genoscope.cns.fr/microscope/home/index.php).

### Antibody generation and immunoblotting

The putative anti-WP_009060351 antibodies were generated using protein bands cut out of SDS-PAGE gels. The band corresponding to a protein of approximately 25 kDa was cut out and sent to Eurogentec (Belgium) for the immunization of rabbits. To confirm if the antibody was raised against the putative lipoprotein, blots were made from 4 to 16% SDS-PAGE gels containing purified expressed proteins as described above. Then the antibody was used for blots from the SDS-PAGE gels containing total crude extract, membrane proteins, lipoproteins and peptidoglycan from *M. fumariolicum* SolV. Immunoblotting was performed on blots incubated in deionized water (ddH_2_O) for 30 min, followed by 30 min incubation in protein-free (Tris-buffered saline [TBS]) blocking buffer (Thermo Scientific, Rockford, IL, USA). Blots were then incubated for 60 min in antiserum diluted 1,000-fold in blocking buffer. Two negative controls were performed; one was incubated in blocking buffer instead of antiserum, and the other was incubated in pre-immune serum diluted 1,000-fold in blocking buffer. Blots were then washed three times for 10 min in TBS containing 0.05% Tween and incubated for 60 min with monoclonal mouse anti-rabbit IgG alkaline phosphatase conjugate (Sigma, Zwijndrecht, The Netherlands) diluted 150,000-fold in blocking buffer. Blots were then washed twice for 10 min in TBS containing 0.05% Tween and twice for 10 min in 10 mM TBS containing 8% NaCl and 0.2% KCl, and finally incubated with a 5-bromo-4-chloro-3-indolylphosphate (BCIP)/nitroblue tetrazolium (NBT) liquid substrate system (Sigma, Zwijndrecht, The Netherlands) for 9 min and rinsed for 10 min in ddH_2_O.

### Bioinformatic analysis

The most updated version of the genome from strain SolV is available at NCBI/Genbank under accession number OX458932.1 The amino acid sequence of protein WP_009060351 (translated from its encoding gene *MFUM*_0397) from *Methylacidiphilum* strain SolV (taxid:1,156,937) used in a BlastP search against the genome of *Methylacidiphilum* strains: Kam1 (taxid:1,202,785), Yel (taxid:1,847,730), Phi (taxid:1,847,729) and V4 (taxid:481,448)), *Methylacidmicrobium* strains: AP8 (taxid:2,730,359), 4AC (taxid:1,041,768), 3C (taxid:1,134,055), LP2A (taxid: 2,796,139) and 3B (taxid:1,041,766), and metagenome assembled genomes (MAGs) from ‘Candidatus *Methylacidithermus pantelleriae* PQ17’ (taxid:2,744,239) and a Verrucomicrobiota subdivision 6 bacterium (taxid:1,655,590). The BlastP parameters were: Expect treshold 0.05; Word size 5; Matric Blosum62; Existence: 11 / Extension: 1; Conditional compositional score matrix adjustment. Sequences were aligned by the Muscle tool and MEGA7 was used to infer the evolutionary relationships of the protein sequences using the Neighbor-Joining method. Bootstrapping involved 500 replications (Kumar et al. 2016). SignalP 6.0 (Teufel et al. 2022) and PRED-LIPO (Bagos et al. 2008) software was used to evaluate signal peptides. The ExPASy tool (http://web.expasy.org/compute_pi/) was used to calculate the total number of amino acids, molecular weight, theoretical pI and peptide mass. PSIpred (http://bioinf.cs.ucl.ac.uk/psipred) was used to provide secondary structure prediction McGuffin et al. 2000). AlphaFold2 (Jumper et al. 2021) was used to predict the protein structure (https://colab.research.google.com/github/sokrypton/ColabFold/blob/main/AlphaFold2.ipynb) and structure visualizations were created in Pymol. TMHMM-2.0 software was used to predict transmembrane helices. Multiple sequence alignment was displayed using ENDscript 2.0 (Robert and Gouet 2014).

## Results

### Transcriptome analysis

**Fig. 1:**
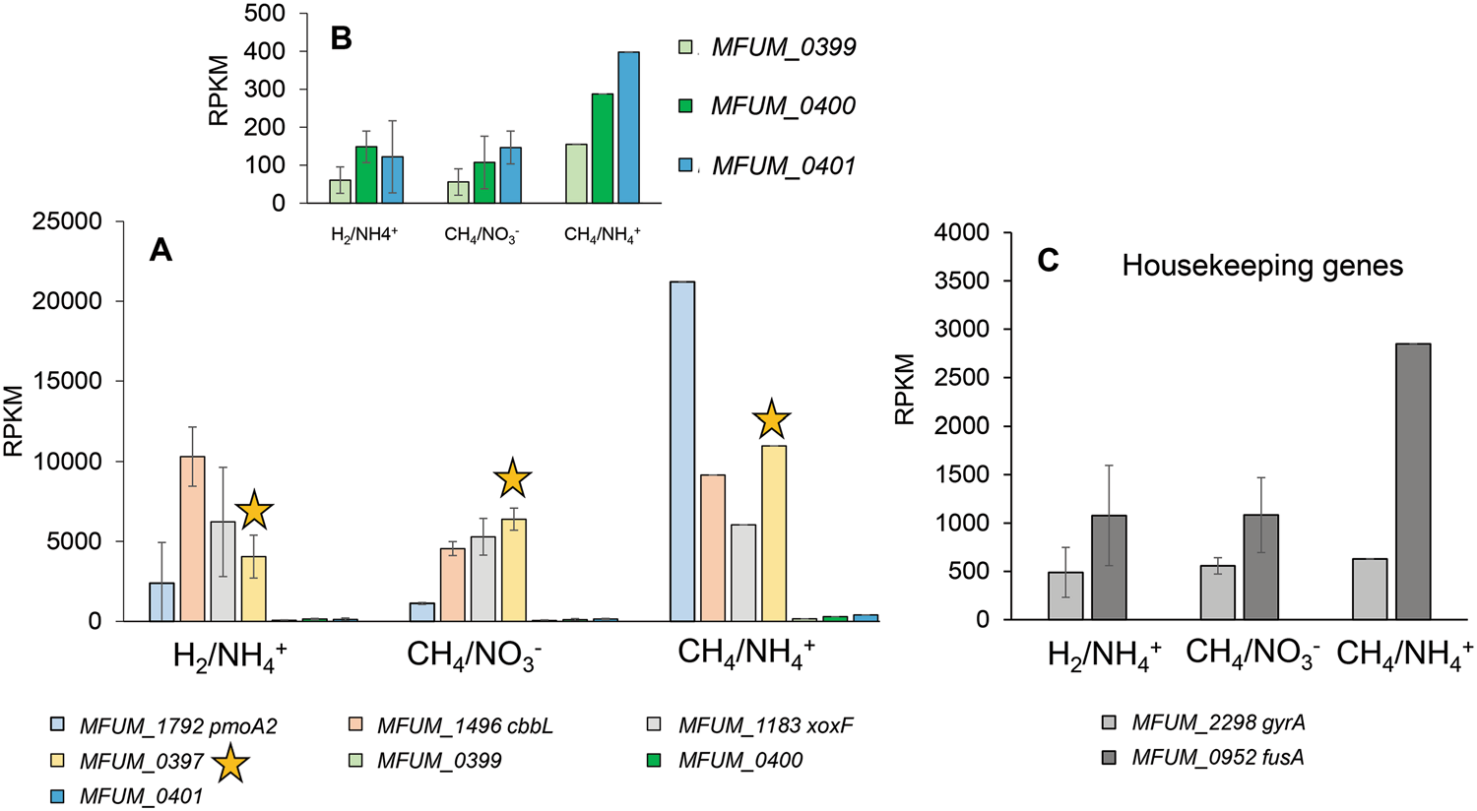
Gene expression (RNAseq) in *M. fumariolicum* SolV. **A** Expression values reported as RPKM of *MFUM*_1792 (*pmoA2*), *MFUM*_1496 (*cbbL*), *MFUM*_1183 (*xoxF*), *MFUM*_0397, *MFUM*_0399, *MFUM*_0400, *MFUM*_0401 (*galT*) in cells grown at the maximum growth rate (CH_4_/NH_4_^+^), and in a chemostat under CH_4_/NO_3_^+^ and H_2_/NH_4_^+^ conditions. **B** Zoomed in on expression values of the three genes flanking *MFUM*_0397 in the same gene cluster. **C** For comparison, expression values reported as RPKM of housekeeping genes *gyrA* and *fusA*. Data from Mohammadi et al. 2017

Analysis of transcriptomic data from *M. fumariolicum* SolV cells growing at maximum growth rate revealed a variety of genes to be highly expressed (Mohammadi et al. 2017). This included notably the key genes involved in methane oxidation (such as *pmoABC* and *xoxF*), and the CBB cycle for carbon fixation (such as *cbbL*/*cbbS*) (Fig. 1A). Surprisingly, the gene *MFUM*_0397 encoding the putative lipoprotein WP_009060351 was expressed at levels as high as those encoding the key metabolic proteins mentioned above (Fig. 1A). Remarkably the preceeding genes *MFUM_0399-401* showed 10–50-fold lower expression levels (Fig. 1B) pointing to *MFUM*_0397 being a single gene rather than part of an operon. The distance between the start of *MFUM_0397* and the end of *MFUM_0399* is 44 bases. For comparison expression of two housekeeping genes are shown (Fig. 1C). In addition, the high expression of this lipoprotein WP_009060351 was also found under other cultivation conditions including cells growing in a chemostat under CH_4_/NO_3_^−^ and H_2_/NH_4_^+^ conditions (Mohammadi et al. 2017). The divergence seen between the three culture conditions relates to the different growth rates at these conditions.

### Bioinformatic analysis of lipoprotein WP_009060351 and its putative processing proteins

**Fig. 2:**
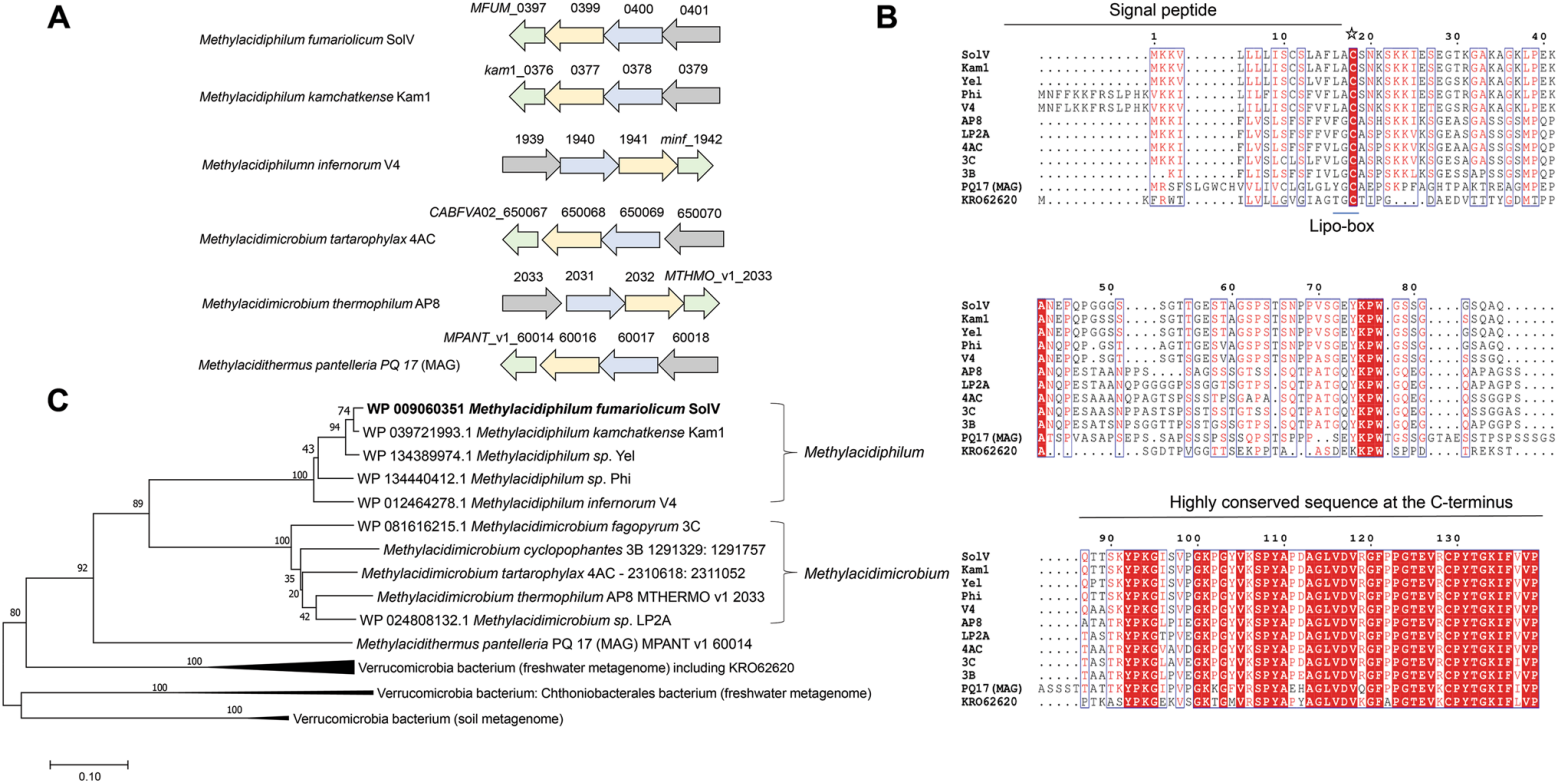
Protein sequence analysis and alignment of WP_009060351 with its orthologous. **A** The operon of *MFUM*_0397 in the *Methylacidiphilum* strains SolV, Kam1 and V4, *Methylacidimicrobium* strains 4AC and AP8 and in the metagenome assembled genome (MAGs) from ‘Candidatus *Methylacidithermus pantelleriae* PQ17’. The different parts of the operon are annotated as follows: (i) *MFUM*_0397, encoding a predicted lipoprotein of 14 kDa (the target of this study, in green); (ii) *MFUM*_0399, encoding a putative DUP domain protein (24.22% identity to *MFUM*_0400, in yellow); (iii) *MFUM*_0400 encoding α-amylase (*amyA,* in blue); (iv) *MFUM*_0401, encoding galactose-1-phosphate uridyl transferase (galT, in gray). **B** Homologous analysis of amino acid sequences. Aligned amino acid residues conserved in all proteins are highlighted in red. Signal peptide regions and highly conserved regions are marked by lines, and the stars indicate the important amino acids. **C** Phylogenetic analysis of WP_009060351 and its homologs based on the protein sequences

WP_009060351 contains 139 amino acids, of which 17 residues MKKVLLLLISCSLAFLA at its N-terminus were predicted to be a lipoprotein signal peptide with high confidence (99.9%) by SignalP 6.0 and PRED-LIPO analyses. The predicted molecular masses are 14,181 Da with and 12,342 Da without the signal peptide, and the theoretical isoelectric point is 9.49 (ExPASy tool). This lipoprotein is composed of 53% nonpolar and 24% polar amino acids. In addition, secondary structure elements were predicted by PSIpred, and a three-dimensional structure was predicted using AlphaFold2 (Supplementary Figure S1). The lipoprotein sequence was used in a BlastP search to find homologs/orthologs in the GenBank nr database (Supplementary Table S2). Orthologous proteins from *Methylacidiphilum* strains (Kam 1, Yel, Phi and V4), *Methylacidmicrobium* strains (AP8, 4AC, 3C, LP2A and 3B) and metagenome assembled genomes (MAGs) from ‘Candidatus *Methylacidithermus pantelleriae* PQ17’ (Picone et al. 2021) and a Verrucomicrobiota subdivision 6 bacterium (KRO62620) were selected and aligned (Fig. 2A, B). SignalP 6.0 analysis of the aligned proteins showed that they all had a hydrophobic lipoprotein signal peptide (Sec/SPII) at the N-terminus. Proteins from *Methylacidiphilum* strains (SolV, Kam 1, Yel, Phi and V4) have a traditional lipobox motif “LAC”, while the others seem to have a deviating lipobox motif “[FLYT]GC”. Remarkably, all proteins have a typical motif at the C-terminus, comprising 53 amino acids, which is widely distributed in verrucomicrobial (meta)genomes only and highly conserved. Subsequently, a phylogenetic tree was built based on homologous proteins and the result revealed the evolutionary relationship between WP_009060351 and its orthologues (Fig. 2C). WP_009060351 from *M. fumariolicum* SolV is most closely related to the lipoproteins from *Methylacidiphilum sp.* Kam1 and Yel. The phylogeny of WP_009060351 with the four verrucomicrobial genera is consistent with a phylogenetic tree of 16S rRNA gene sequences (see Picone et al. 2021).

After synthesis of lipoprotein WP_009060351 in the cytoplasm, the Sec/SPII type signal peptide (see above) will ensure translocation across the IM. Further maturation of the prelipoprotein involves its lipidation by the proteins encoded by the genes *lgt*, *lspA* and *lnt* (see Introduction). Based on homologous genes from *E. coli*, genes for lipoprotein maturation in *M. fumariolicum* SolV, *MFUM*_0361, *MFUM*_0005, and *MFUM*_0851 were predicted to encode Lgt, Lsp and Lnt, respectively (Table 1).

**Table 1:** Gene predictions in *Methylacidiphilum fumariolicum* SolV

Encoded function	Gene ID SolV	Protein identity	BlastP E-value	Domain E-value
Phosphatidylglycerol—prolipoprotein diacylglyceryl transferase Lgt	MFUM_0361	39%	4e-54	8e-75
Lipoprotein signal peptidase LspA	MFUM_0005	32%	1e-18	7e-36
Apolipoprotein N-acyltransferase Lnt	MFUM_0851	27%	3e-32	9e-89
*Lipoprotein release complex I LolE2D2*				
Inner membrane subunit LolE	MFUM_2245	28%	3e-44	1e-98
ATP binding subunit LolD	MFUM_2246	44%	3e-61	3e-92
*Lipoprotein release complex II LolCED2*				
Inner membrane subunit infusion LolCE	MFUM_2483	24%	7e-07	4e-22
ATP binding subunit LolD	MFUM_2484	48%	2e-67	2e-114
Periplasmic chaperone SurA	MFUM_2159	24%	1e-12	5e-26
Periplasmic serine endoprotease DegP	MFUM_2004	41%	3e-90	3e-175
Periplasmic chaperone Skp	MFUM_1850	23%	3e-06	8e-19
Outer membrane protein BamA	MFUM_1849	25%	1e-59	3e-164
BamD*	MFUM_1939	36%	3e-68	2e-22

The amino acid sequences of Lgt (CAD6004980), LspA (.CAD6022384), Lnt (CAD6019362), LolC/E (CAD6016608), LolD (EFI21389), SurA (CAD6022301), DegP (CAD6021993), Skp (CAD6021923) and BamA (VWQ00961) from *E. coli* K-12 were retrieved from GenBank and used for BlastP searches against *M. fumariolicum* SolV genome. BamD*, BamD (VWQ04786) from *E. coli* was used for blasting against the Verrucomicrobiota (NCBI:txid2026799), the best hit BamD from Chthoniobacterales bacterium was used for a second Blast search against *M. fumariolicum* SolV genome. After identification the proteins of SolV were used in a domain search for confirmation.

To transfer mature lipoproteins from the IM to the inner leaflet of the OM, the localization of lipoprotein (lol) pathway is used (Okuda and Tokuda 2011; Sharma et al. 2021). This process is dependent on the ATP-binding cassette (ABC) transporter LolCDE which captures and extrudes the lipoproteins from the IM. LolA shuttles the lipoprotein from the IM to the OM, and the OM lipoprotein receptor LolB plays a critical role in the incorporation of lipoproteins in the OM. BlastP searches revealed the presence of two distinct *lolD* genes (*MFUM*_2246 and *MFUM*_2483, identity: 41.70%), a *lolE* gene (*MFUM*_2245) and a putative fusion *lolCE* gene (*MFUM*_2483), whereas *lolA* and *lolB* genes were absent (Table 1). In general, this was the case for all available genomes of *Methylacidiphilum*, *Methylacidmicrobium*, and *Methylacidithermus* species while available metagenomes of other members of the phylum Verrucomicrobiota do contain LolA and lack LolB. Homologs encoding SurA, DegP, Skp, BamA and BamD are present in the genome and may act as alternative for LolA and LolB (see Discussion).

Besides amino acids analysis, the gene cluster of *MFUM*_0397 (encoding WP_009060351) and its orthologues were analyzed (Fig. 2A). Although the gene *MFUM*_0397 seems to be located in a polycistronic operon with three other genes, transcriptome data (see above) point to *MFUM*_0397 being a single gene rather than part of an operon. The other proteins encoded at close distance seem to be involved in glycosylation/deglycosylation reactions since they encoded two putative α-amylases and a putative galactose-1-phosphate uridyl transferase. *MFUM*The gene sequences similar to *MFUM*_0397-0401 are in a comparable organisation and conserved in *Methylacidiphilum* species Kam1 and V4, *Methylacidimicrobium* species 4AC and AP8, and ‘Candidatus *Methylacidithermus pantelleriae* PQ17’ (MAG).

### Protein purification and subunit identification

**Fig. 3:**
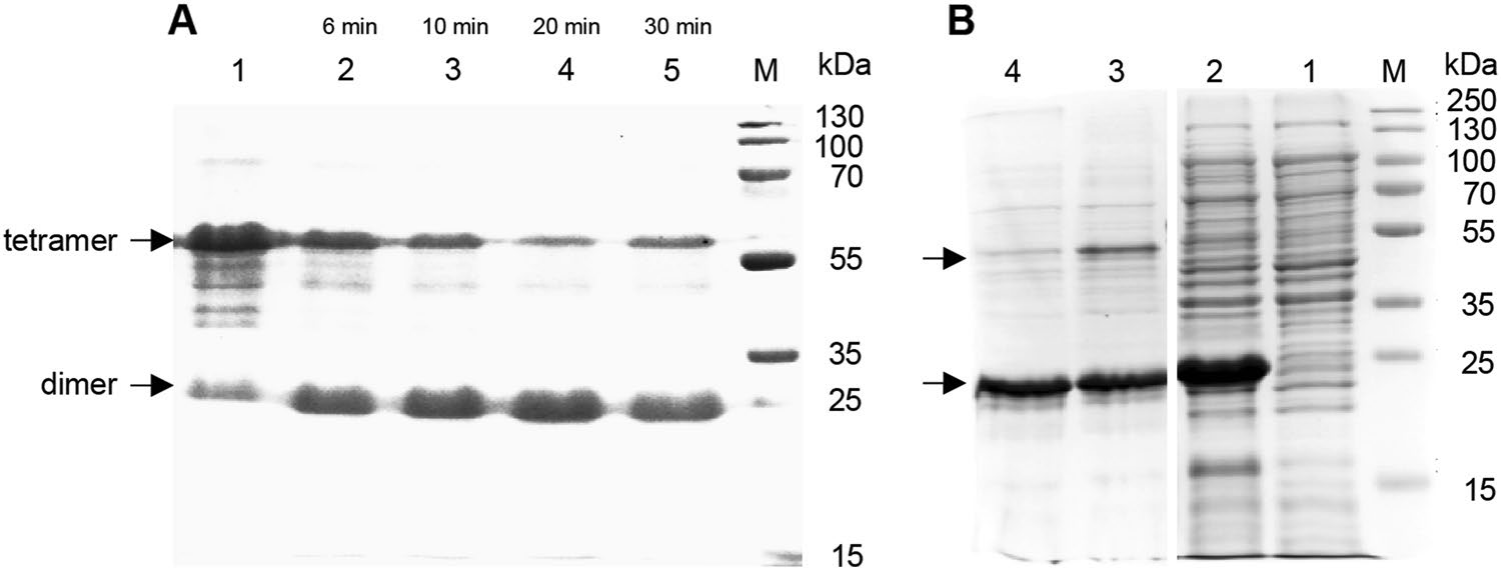
SDS-PAGE gels of the purified recombinant WP_009060351 from *E. coli*. **A** Lanes 1 to 5: purified recombinant WP_009060351 mixed with sample buffer and heated at 100 °C for 0, 5, 10, 20, and 30 min, respectively before running the gel; **B** TritonX-114-purified protein WP_009060351 (no His-tag) from *E. coli*. The pellet (lane 1) and supernatant (lane 2) fractions of *E. coli* cells after centrifugation at 10,000 × g at 4 °C for 20 min, mixed with sample buffer, and boiled for 10 min at 100 °C.; Triton X-114-purified protein with, mixed with sample buffer at room temperature (lane 3) and boiled for 10 min at 100 °C (lane 4) min

A preliminary experiment was performed using a Triton X-114 phase partitioning method to extract lipoproteins from *M. fumariolicum* SolV cells according to the enrichment of bacterial lipoproteins protocol (Armbruster and Meredith 2018). Unfortunately, lipoprotein WP_009060351 was not detected by SDS-PAGE and MALDI-TOF MS analysis. Thereafter the His-tagged lipoprotein WP_009060351 was heterologously overexpressed in *E. coli* and purified using a Ni–NTA resin. As shown by SDS-PAGE analysis (Fig. 3A), two bands at around 25 kDa and 60 kDa were found, which were both unambiguously identified as WP_009060351 by MALDI-TOF MS analysis after tryptic digestion. Based on the analysis of the size of bands, we postulated bands at around 25 kDa and 60 kDa might be dimers and tetramers of WP_009060351, respectively. Apparently these multimers existed despite the SDS treatment. To confirm our hypothesis, purified WP_009060351-His tag protein was incubated in SDS sample buffer at two temperatures and different periods of time. After cooling immediately on ice water, the samples were loaded on SDS-PAGE gel. From Fig. 3A, the sample mixed with SDS sample buffer at room temperature showed a thick band at around 60 kDa and a less intense band at around 25 kDa (Fig. 3A; Lane 1). With the temperature increased to 100 °C and boiling times from 5 to 30 min, the band at around 60 kDa shifted into a more dominant 25 kDa band (Lanes 2–5). To determine if the putative dimer protein could be converted into the monomer, the purified sample was treated under different harsh conditions, including heating in a block up to 150 °C for 20 min, acid pH 3 for 30 min and long-time boiling (up to 1 h). No monomeric form of the protein was observed. Based on these results, the recombinant lipoprotein in *E. coli* seems to be present in two forms: a dimer and a tetramer. The tetramer is probably the predominant form in *E. coli* cells, however, the tetramer can be converted into a stable dimer. *E. coli* cells may perform some post-translational modification of the expressed protein (e.g. glycosylation) affecting the in vivo appearance. Glycan-detecting periodic acid Schiff’s (PAS) staining on an SDS-PAGE gel of the native protein showed no bands. An alternative explanation could be that lipidation and covalent binding would affect the behaviour of heterologous expressed protein on SDS-PAGE. To investigate if lipoprotein WP_009060351 expression in *E. coli* would affect the IM and OM stability, preliminary diSC3(5) release assays and NPN uptake assays were performed with a plasmid-containing MFUM_0397 and a control (empty plasmid pET-30a). The results showed that the expression of WP_009060351 did not result in morphological changes and had no effect on the OM. Only a slight increase of the inner membrane potential gradient was observed (Supplementary Figure S2).

After treatment with Triton X-114, the recombinant lipoprotein (His-tag) was in the supernatant (Fig. 3B, Lane 2), not in the pellet (Fig. 3B, Lane 1). Identical results were found with Triton X-114-extracted recombinant lipoprotein without His-tag. This is consistent with lipid modification of the recombinant protein, two bands at around 25 kDa and 60 kDa (no His-tag) were shown in a sample without boiling before running SDS-PAGE (Fig. 3B, Lane 3), while only one band at around 25 kDa was present after boiling (Fig. 3B, Lane 4).

### Immunoblotting

The band at around 25 kDa that was obtained from recombinant protein with His-tag purified from *E. coli* and confirmed by MALDI-TOF analysis to be protein WP_009060351 was used to immunize a rabbit to generate anti-WP_009060351. Considering the recombinant protein containing a 6x-His tag and to exclude tag effects on the result, we examined the genome of *M. fumariolicum* SolV and no 6xHis motifs were found. Additionally, InVision™ His-tag In-gel Stain (Thermo Fisher Scientific) was used to verify the presence of 6x-His in the total crude extract of *M. fumariolicum* SolV and no bands were observed.

**Fig. 4:**
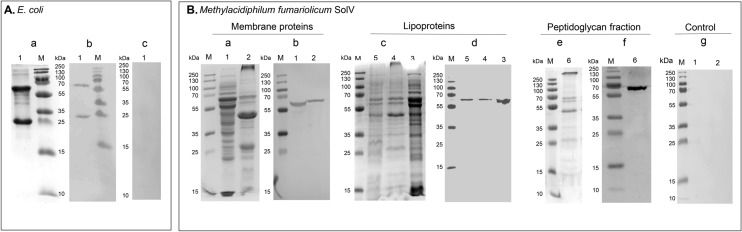
Immunoblot analysis of the purified proteins from *E. coli* and different fractions from *M. fumariolicum* SolV. **A** Purified lipoprotein WP_009060351-His-tag from *E. coli.* **a**, protein sample on SDS-PAGE, Coomassie stained; **b**, immunoblots were incubated with anti-WP_009060351; **c**, immunoblots were incubated with preimmune serum. **B** Fractions extracted from strain SolV. **a**, **c**, **e**: protein samples separated on SDS-PAGE, Coomassie stained; **b**, **d**, **f**: immunoblots were incubated with anti-WP_009060351; **g**: immunoblots were incubated with preimmune serum. Lanes 1, represent total soluble protein from strain SolV; Lanes 2, represent total membrane proteins from strain SolV; Lanes 3, represent protein in aqueous phase from strain SolV; Lanes 4, represent protein in Triton X-114 phase from strain SolV; Lanes 5, represent protein in pellet from strain SolV; Lanes 6, represent protein in peptidoglycan fraction from strain SolV

Incubation of blotted purified recombinant proteins with the obtained antisera resulted in a positive immunoblot reaction (Fig. 4A. b). Controls with preimmune serum of the immunized rabbits showed no reaction (Fig. 4A. c). Samples isolated from *M. fumariolicum* SolV cells and representing total membrane proteins (Fig. 4B. ab), lipoproteins (Fig. 4B, cd), and peptidoglycan fractions (Fig. 4B. e, f) all reacted to the polyclonal antibody raised against anti-WP_009060351 showing a single band at about 60 kDa. This also points to a tetrameric (most likely lipidated) appearance like in *E.* coli cells expressing the protein (see above). The higher molecular mass could also be caused by lipidation or covalent linkages of one or more monomers”. The soluble fraction also showed a band which could be expected for a highly expressed protein that is first synthesised in the cytoplasm. Controls using preimmune serum of the immunized rabbits did not show any reactivity (Fig. 4B. g). An attempt to identify the band by MALDI-TOF MS, was not successful. To confirm protein localization in *M. fumariolicum* SolV, immunogold localization with transmission electron microscopy (TEM) was performed. Unfortunately, anti-WP_009060351 did not work. The results of immunoblotting suggest that WP_009060351 might be an SDS-resistant tetramer in *M. fumariolicum* SolV cells and present in the membrane, peptidoglycan, and cytoplasm fractions.

## Discussion

In this study, we have identified a highly expressed lipoprotein (WP_009060351) in the verrucomicrobial methanotroph *M. fumariolicum* SolV. The first 90 amino acids of WP_009060351 are specific for the genera *Methylacidiphilum*, *Methylacidimicrobium*, and *Methylacidithermus* and may have specific functions relative to their volcanic habitat. The last 53 amino acids of WP_009060351 appear to represent a domain that is widely distributed and highly conserved in verrucomicrobial (meta)genomes but not present in members of other phyla, and the encoded proteins all possess the typical cysteine at the N-terminus after cleavage of the signal peptide.

Considering a specific niche within the geothermal environment with low pH and high temperature, verrucomicrobial methanotrophs produce predominantly (> 96%) saturated phospholipids to regulate membrane permeability (van Teeseling et al. 2014a). Specifically, the *M. fumariolicum* SolV fatty acid composition shows 42.1% stearic acid (18:0), 24.5% 12-methyltetradecanoic acid (a15:0), 11.6% 12-methyltridecanoic acid (i14:0) and 10.5% palmitic acid (16:0). The lipid moiety of the lipoproteins is derived from membrane phospholipids. Further studies including radioactive labelling of fatty acids, such as [^3^H]-palmitic acid could be used to confirm lipidation of WP_009060351. Glycosylation could be an additional posttranslational modification for the lipoprotein (Ristl et al. 2011) but was not observed for the native protein.

Protein localization plays an important role in characterising the cellular functions of hypothetical and newly discovered proteins. A positive immunoblot reaction of WP_009060351 was obtained following incubation of total membrane proteins, a lipoprotein enrichment fraction, and a peptidoglycan fraction from *M. fumariolicum* SolV with the specific antiserum. Unfortunately, this antibody did not work in the TEM immunogold localization. Lipoprotein maturation and trafficking in *E. coli* has been gradually unravelled. The three genes encoding the proteins Lgt, Lsp and Lnt known to be involved in maturation (acylation) of the pre-lipoprotein could be identified in the genome of strain SolV (Table 1). After lipid modification and processing, mature lipoproteins are localized to either the IM or the OM depending on the sorting signals in diderm (Gram-negative) bacteria. Studies showed the presence of an Asp at position + 2 leads to retaining lipoprotein in the IM, while Ser or another residue at that position causes an outer membrane localization of this lipoprotein (Seydel et al. 1999; Yamaguchi et al. 1988). This rule is called the “ + 2 rule” and it is generally conserved in *Enterobacteria*. In the case of lipoprotein WP_009060351 (N-terminus CSNK), the Ser residue present at position + 2 functions as the OM sorting signal, and therefore WP_009060351 is likely to accumulate in the OM. However, the “ + 2 rule” does not always apply for all species (Lewenza et al. 2008; Seydel et al. 1999). For example, in *Pseudomonas aeruginosa* amino acids at positions + 3 and + 4 also play a critical role in lipoprotein sorting (Lewenza et al. 2008; Nishimura et al. 2011).

**Fig. 5:**
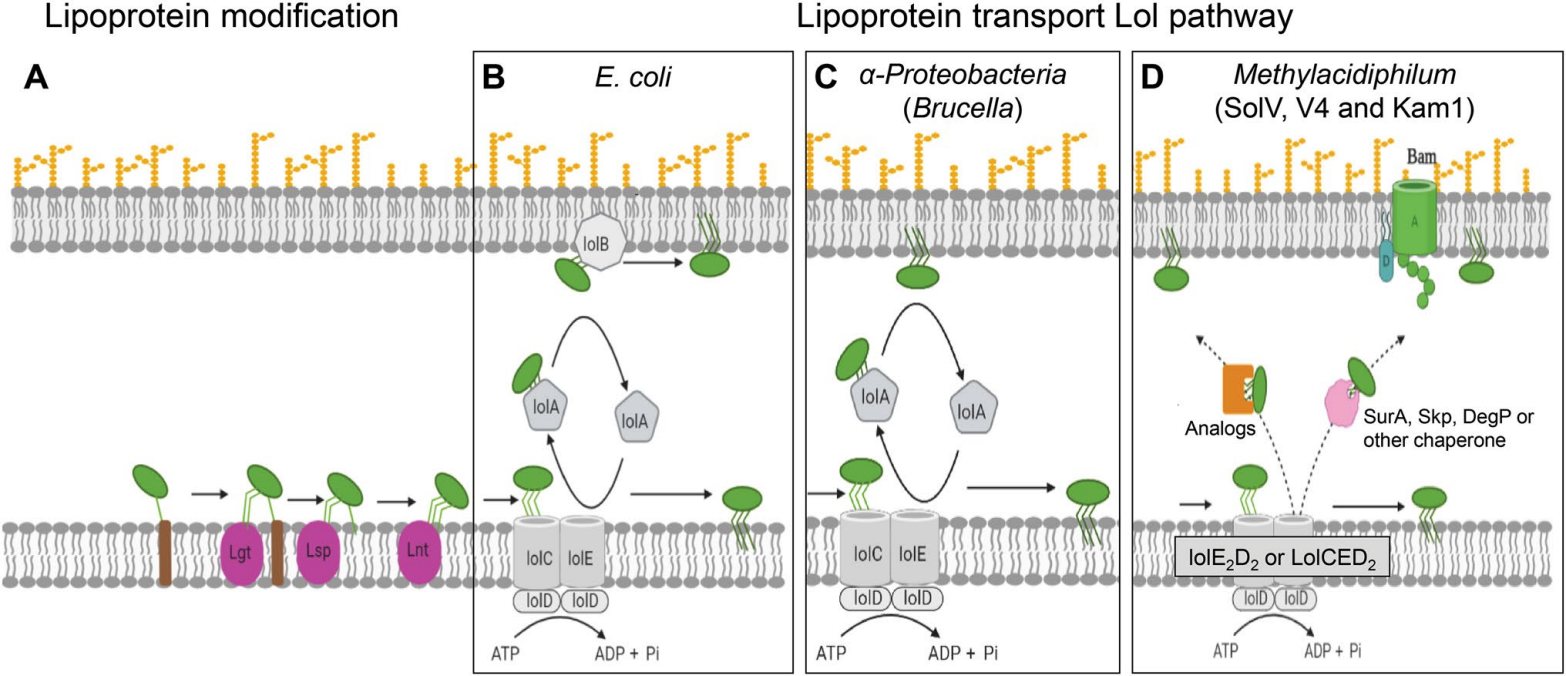
Model of lipoprotein modification and lipoprotein transport. **A** Conversion of prelipoproteins to mature lipoprotein catalyzed by three enzymes Lgt, Lsp and Lnt. Lipoprotein (green) and its signal peptide (brown). **B** Lol pathway in *E. coli*. Lol pathway is composed of ABC transporter complex LolCED_2_, carrier protein LolA and OM lipoprotein receptor LolB. **C** Lol pathway in a *Brucella* sp. Lol pathway is consist of LolCED_2_ and LolA, LolB is absent. LolA plays a role as a carrier protein in the localization of lipoproteins, but also in the insertion of lipoproteins into the OM. **D** Putative Lol pathway in *Methylacidiphilum* strains SolV, V4 and Kam1. Two ABC transporter LolE_2_D_2_ and LolCED_2_ were predicted. These strains lack both LolA and LolB, functional analogs unrelated to the LolA and LolB sequences, or periplasmic chaperones such as SurA, Skp and DegP from the Bam complex may be used to transport lipoproteins to OM, where the Bam complex inserts them into the inner leaflet of OM. Abbreviations used: diacylglyceryl transferase (Lgt), lipoprotein signal peptidase II (Lsp), lipoprotein N-acetyltransferase (Lnt), inner membrane (IM), lipoprotein localization machinery (Lol), lipoprotein release complex—inner membrane subunit (LolC and LolE), lipoprotein release complex—ATP binding subunit (LolD), carrier protein (LolA), OM lipoprotein receptor (LolB), adenosine triphosphate (ATP), outer membrane (OM), ATP binding cassette (ABC), β-barrel assembly machinery (Bam complex) BamA and BamD

In *E. coli*, lipoproteins are transferred from IM to the OM via the Lol pathway (Terada et al. 2001; Okuda and Tokuda 2011; Konovalova et al. 2015). This pathway uses an ABC transporter (LolCDE) to release lipoprotein from the IM, a carrier protein LolA to shuttle cargo from the IM to the OM lipoprotein receptor LolB, which mediates lipoprotein integration into the OM (Fig. 5). The LolCDE complex is composed of a heterodimer of the membrane proteins LolC and LolE, and a homodimer of an ATPase subunit—LolD. LolC and LolE exhibit 26% sequence identity to each other and also have similar membrane topologies (Yasuda et al. 2009). Both LolC and LolE have 4 transmembrane spans. The protein MFUM_2483 from strain SolV contains the homologous sequence to both LolC and LolE. TMHMM-2.0 predicted ten transmembrane segments, which is similar to most ABC transporters. Therefore, we speculate that *MFUM*_2483 probably encodes a fusion protein of LolC and LolE, and with *MFUM*_2484 encoding LolD this could form a LolCED_2_ complex. Furthermore, *MFUM*_2245 and *MFUM*_2246 encoding additional separate LolE and LolD which could therefore form a LolE_2_D_2_ complex. These two Lol complexes require protein structure confirmation and it is uncertain which complexes could be specific for the lipoprotein WP_009060351. Neither of genes encoding both LolA and LolB was found in *Methylacidiphilum*, *Methylacidmicrobium* and *Methylacidithermus* species, while in the remaining metagenomes of the other Verrucomicrobiota only LolA was present and LolB was absent. LolB was already reported to be absent in α-Proteobacteria such as *Brucella* sp (Goolab et al. 2015). Since LolA and LolB are homologous to each other, LolA was assumed to play a more extensive role in the lipoprotein localization, inserting lipoproteins into the OM but also acting as a carrier protein (Goolab et al. 2015). Based on current knowledge, we speculate that verrucomicrobial methanotrophs may use functional analogues unrelated to the LolA and LolB sequences or borrow periplasmic chaperones such as SurA, Skp and DegP from the Bam complex (β-barrel assembly machinery) to transport lipoprotein to the OM, and the Bam complex inserts it in the inner leaflet of the OM. The periplasmic chaperone SurA has been shown to transport nascent outer membrane proteins (OMPs) across the periplasmic space to the OM, and two other periplasmic proteins (Skp and DegP) have been shown to compensate for the absence of SurA and may be more important for dealing with proteins that have fallen out of the efficient assembly pathway (Hagan et al. 2011; Sklar et al. 2007). Homologous of periplasmic chaperone SurA, Skp and DegP and Bam complex components BamA and BamD were found in *M. fumariolicum* SolV (Table 1), while Bam complex components BamB and BamC were absent. How this Bam complex works remains to be studied.

Based on the " + 2 rule" described above, it is likely that WP_009060351 accumulates in the OM. Recently, lipoproteins have also been reported to be exposed on the cell surface in relation to pathogenesis, in addition to being located on the IM and OM (Wilson and Bernstein 2016). Clearly, identification of the lipoprotein biosynthetic pathway in new strains remains an important area for future work.

Since no genetic system is available for *M. fumariolicum* SolV, it is not possible to make knockout mutant of the lipoprotein encoding gene to assess its function. Protein localization, high expression level, and knowledge of abundant lipoproteins in other bacteria may help to predict the function of this lipoprotein. Tethering of the OM to the cell wall in diderm (Gram-negative) bacteria, is essential to preserve cell envelope integrity (Witwinowski et al. 2022). Three different systems of OM tethering are identified; Lpp, Pal and OmpA with OmpM as an alternative system reported from some members of the superphylum Terrabacteria. The peptidoglycan-associated lipoprotein (Pal) system is restricted to the diderm bacteria, along with sporadic occurrence of the OmpA system. The Lpp system is present only in a subclade of the Pseudomonadota. Corresponding genes of the tethering systems were used in a BlastN search against the genome of *M. fumariolicum* SolV, showing the presence of the genes *pal* and *ompA*. Expression levels of these genes were 20-fold compared to *MFUM_0397*. No homologous *lpp* gene was found in *Methylacidiphilum* and *Methylacidmicrobium* species. Lpp, also known as Braun’s lipoprotein or murein lipoprotein, is the first identified lipoprotein attached to a membrane by a lipid moiety (Hayashi and Wu 1990). It is a small alpha-helical lipoprotein (5.8 kDa) that is considered to be the most abundant (lipo)protein in *E. coli*, with more than 1 million copies per cell (Li et al. 2014; Mathélie-Guinlet et al. 2020). In terms of the massive expression of Lpp, several interesting features were highlighted. First is the promoter region of the *lpp* gene, which has a high AT content, not only in *E. coli* (Nakamura and Inouye 1979), but also in *Serratia marcescens* (Nakamura and Inouye 1980) and *Erwinia amylovora* (Yamagata et al. 1981). A high AT content promotes the melting of RNA polymerase for transcription initiation, a feature that may be beneficial to the high transcription efficiency of the *lpp* gene (Nakamura and Inouye 1979). Second, the fact that the Lpp mRNA is extraordinarily stable, having a half-life of 11.5 min, which is more than the average mRNA half-life of 1.3 min (Hirashima et al. 1973). We also analyzed the promoter region of the gene cluster in which the lipoprotein WP_009060351 is located. This region, 132 bp in size, also present in strain Kam1 shows a high AT content of 68%, which could be an explanation of the high expression levels achieved. The amino acid sequence of Lpp was aligned with WP_009060351 and its orthologues. This alignment (Supplementary Figure S1), showed three highly conserved amino acids, one is the conserved cysteine (C), which is the first amino acid of the mature lipoprotein after cleaving of the signal peptide, the other two are tyrosine (Y) and lysine (K) at the C-terminus. These three highly conserved amino acids (C, Y, K) are also conserved in a protein encoded in a verrucomicrobial MAG from a freshwater metagenome. The C-terminal lysine (K) residue of Lpp from *E. coli* is covalently attached to the peptidoglycan, providing the only covalent connection between the outer membrane and the cell wall, tethering the OM to the PG and determining the size of the periplasm (Asmar et al. 2017; Cohen et al. 2017; Mathélie-Guinlet et al. 2020. From our results, WP_009060351 was top-ranked among all lipoproteins from *M. fumariolicum* SolV based on mRNA expression levels. Immunoblotting showed WP_009060351 to be present in the isolated peptidoglycan fraction of *M. fumariolicum* SolV. Therefore, we speculate WP_009060351 might be covalently linked with the peptidoglycan, playing a similar role in tethering the OM to the peptidoglycan, adjusting the size of the periplasm in strain SolV. This could be a reason why this high expression lipoprotein was not detected with MALDI-TOF MS in the SDS-PAGE of protein fractions, and why only small amounts were detected in immunoblotting. The latter weak response in the isolated peptidoglycan fraction may be due to β-mercaptoethanol breaking the disulphide bonds of the protein, allowing a small amount of lipoprotein to be released from the peptidoglycan, while a large amount of lipoprotein may still be covalently linked to the peptidoglycan. For studying this lipoprotein, it is important to find ways to break the covalent bond to release the lipoprotein from the peptidoglycan.

## Data Availability

On request raw data supporting the conclusions of this article will be made available by the authors, without any reservation. The most updated version of the genome from strain SolV is available at NCBI/Genbank under accession number OX458932.1 and the identifier MFUM.
